# Primary Central Nervous System Lymphoma Presenting as Optic Nerve Infiltration: A Case Report

**DOI:** 10.7759/cureus.36969

**Published:** 2023-03-31

**Authors:** Therese Franz B Reyes, Rosemarylin Or, Diane Charleen Gochioco

**Affiliations:** 1 Department of Clinical Neurosciences, University of the East Ramon Magsaysay Memorial Medical Center, Quezon City, PHL

**Keywords:** cns lymphoma, optic nerve infiltration, optic nerve involvement, lymphomatous optic nerve infiltration, optic nerve lymphoma, primary cns lymphoma

## Abstract

Primary central nervous system lymphoma (PCNSL) is a rare type of non-Hodgkin lymphoma, which uncommonly presents with optic nerve infiltration (ONI). ONI has been reported mostly in relapse cases of PCNSL and is rarely the sole manifestation of the disease at the time of diagnosis. We report a case of a 69-year-old female who presented with progressive visual impairment with relative afferent pupillary defect (RAPD) on examination. Orbital and cranial magnetic resonance imaging (MRI) revealed bilateral optic nerve sheath contrast enhancement with an incidental finding of a right frontal lobe mass. Routine cerebrospinal fluid analysis and cytology were unremarkable. Excision biopsy of the frontal lobe mass yielded the diagnosis of a diffuse B-cell lymphoma. Intraocular lymphoma was excluded on ophthalmologic workup. Whole body positron emission tomography scan did not reveal extracranial involvement establishing the diagnosis of PCNSL. Chemotherapy was initiated with rituximab, methotrexate, procarbazine, and vincristine as induction regimen and cytarabine as consolidation therapy. On follow-up, the visual acuity of both eyes significantly improved with the resolution of RAPD. Repeat cranial MRI did not show a recurrence of the lymphomatous process.

To the best of the authors’ knowledge, ONI as the initial presentation at the time of PCNSL diagnosis has only been reported three times. The present case’s unusual presentation highlights the need to consider PCNSL as a differential diagnosis in patients who present with visual deterioration and optic nerve involvement. Prompt evaluation and treatment of PCNSL are essential for improving the visual outcomes of patients.

## Introduction

Primary central nervous system lymphoma (PCNSL) is a type of non-Hodgkin lymphoma involving the brain, spinal cord, leptomeninges, proximal parts of cranial nerves, and eyes with no underlying systemic involvement [1]. It typically occurs among immunosuppressed patients. In recent years, its incidence among immunocompetent individuals particularly the elderly population has been increasing [2]. PCNSL typically affects the brain parenchyma with a predilection to the deep and periventricular white matter. Primary symptoms are attributed to the location of the lymphoma. Cognitive impairment, neuropsychiatric symptoms, and focal neurologic deficits are the usual manifestation of the disease [1]. Visual disturbances are reported less frequently. A majority of the documented cases of visual impairment in PCNSL result from an underlying intraocular lymphoma [1]. However, visual symptoms may also arise from underlying optic nerve involvement. This is seen mostly in relapse cases of PCNSL and rarely presents as the only manifestation at the time of diagnosis [3]. We present a case of a 69-year-old female with a visual impairment from an optic nerve infiltration (ONI) as the presenting symptom of PCNSL.

## Case presentation

A 69-year-old female, with a known case of hypertension and type 2 diabetes mellitus, presented to the emergency room with a progressive blurring of vision over a period of two weeks. No complaints of headache, dizziness, cognitive and behavioral changes, signs of infection, or constitutional symptoms were noted before the onset of symptoms. On physical examination, visual acuity of the right eye was 20/200 (0.1) and that of the left eye was 20/800 (0.025). Upon correction, the right eye’s visual acuity improved to 20/100 (0.2), but the left eye remained 20/800 (0.025). Pupillary light reflex examination revealed a relative afferent pupillary defect (RAPD) in the right eye. Fundoscopic findings in both eyes were unremarkable. Motor and sensory examinations were normal.

**Figure 1:**
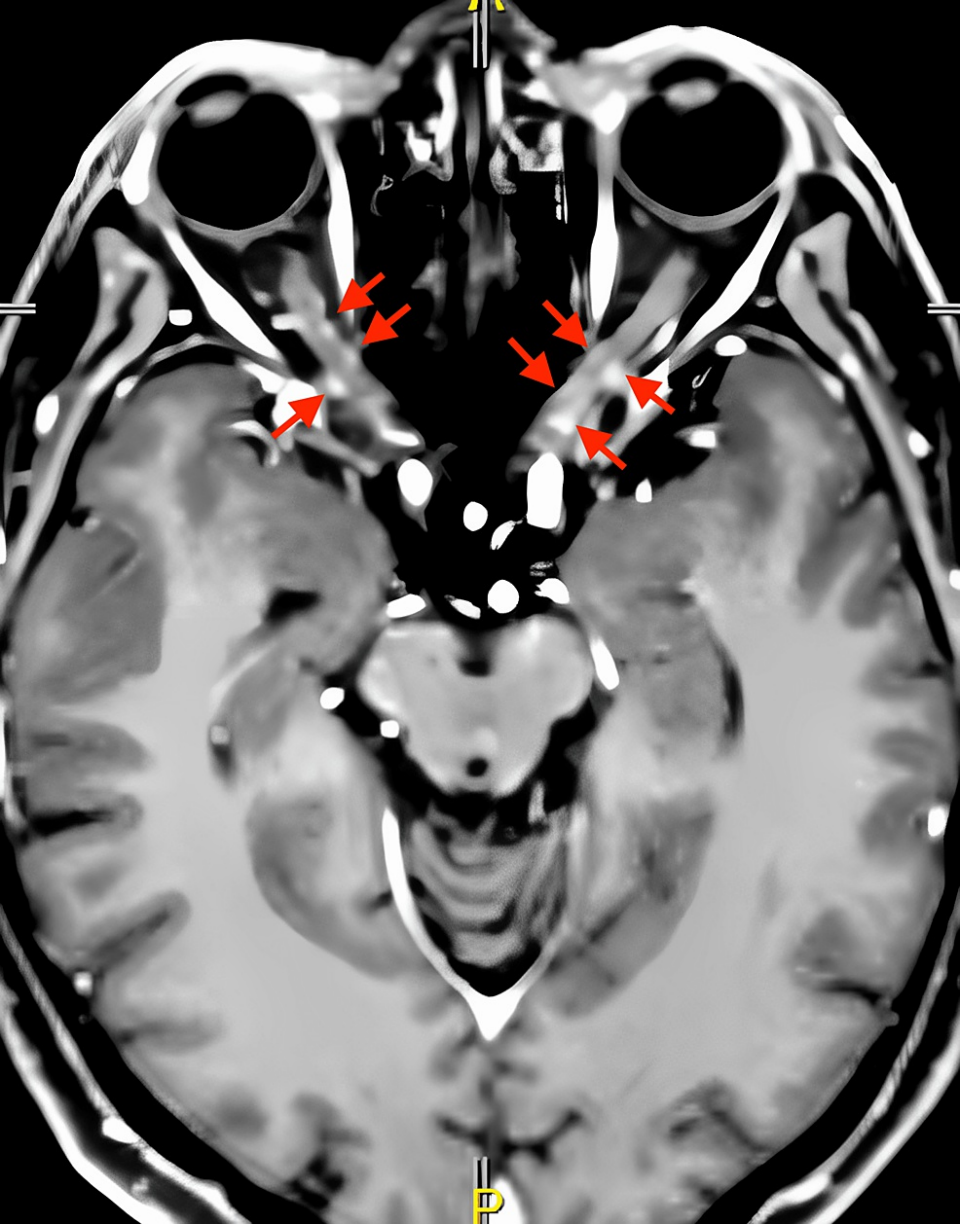
Post-contrast T1-weighted MP-RAGE study showing abnormal enhancement (red arrows) surrounding the bilateral optic nerves MP-RAGE: Magnetization Prepared–RApid Gradient Echo.

**Figure 2:**
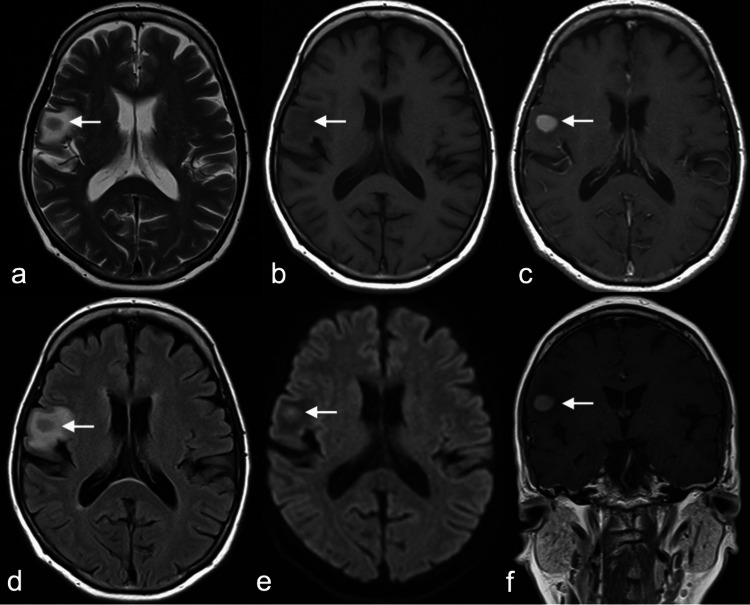
Brain MRI showing the circumscribed lesion involving the right frontal lobe (white arrow) with decreased T2 and T1 signals (a and b). (d) Fluid attenuation inversion recovery image exhibiting mild perilesional vasogenic edema. (e) Diffusion-weighted imaging demonstrating mild restricted diffusion. (c and f) Post-contrast study showing homogenous enhancement of the lesion.

Routine cerebrospinal fluid (CSF) analysis and microbiologic workup were unremarkable. CSF cytology did not reveal malignant cells. Cranio-orbital magnetic resonance imaging (MRI) showed mild abnormal enhancement surrounding the bilateral optic nerves and an incidental finding of a circumscribed enhancing lesion, measuring 1.2 cm x 1.6 cm x 1.2 cm, with mild restricted diffusion over the right frontal lobe (Figures 1, 2).

**Figure 3:**
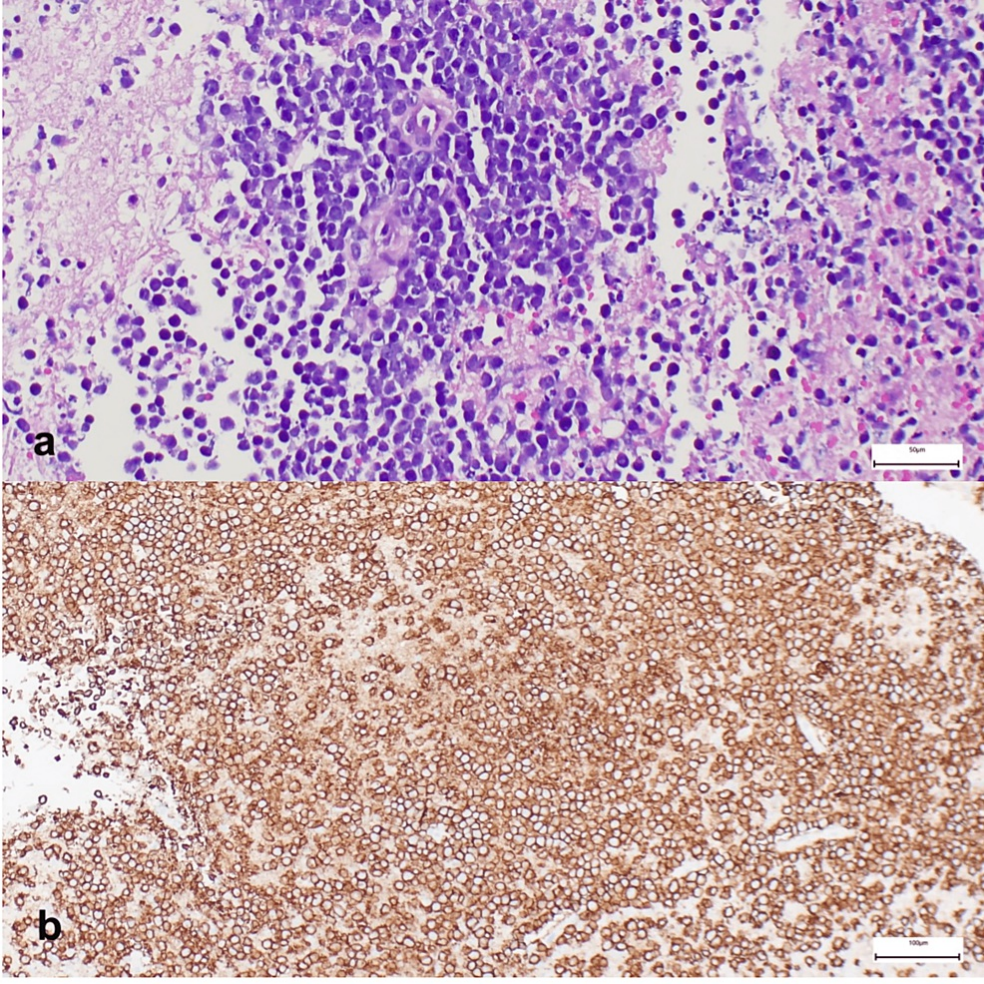
Hematoxylin and eosin stain showing (a) perivascular pattern of large atypical lymphocytes and (b) immunohistochemical stain showing CD20 positivity

Magnetic resonance spectroscopy of the frontal lobe mass only showed a lactate peak, which was non-specific and failed to establish the presence of a definite tumor signature. Excision biopsy of the right frontal lobe mass was immediately done. Histopathology was consistent with lymphoma revealing atypical large round blue cells with scant cytoplasm and prominent nucleoli. Immunohistochemical staining revealed that the tissue was positive for CD20 and negative for CD3, CAM 5.2, and glial fibrillary acidic protein (Figure 3) confirming the diagnosis of a diffuse large B-cell lymphoma.

Whole body positron emission tomography (PET) scan with contrast showed no evidence of an underlying systemic source, which established the diagnosis of a primary central nervous system (CNS) lymphoma. A referral to ophthalmology was made to evaluate for possible intraocular lymphoma. Ophthalmologic workup including fluorescein angiography and macula optical coherence tomography was unremarkable, ruling out an intraocular involvement. Steroid therapy with dexamethasone was started postoperatively. Gradual improvement was noted after steroid initiation with a resolution of RAPD and improvement of visual acuity to 20/50 (0.4) in both eyes.

**Figure 4:**
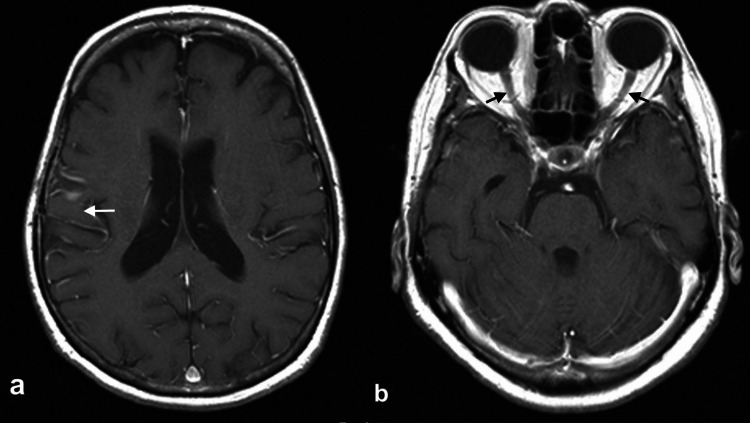
Repeat post-contrast cranial MRI showed (a) no recurrence of intracranial lymphoma (white arrow). (b) Bilateral optic nerves are normal (black arrows).

CSF flow cytometry before chemotherapy was unremarkable. The patient underwent five cycles of the induction phase with rituximab, methotrexate, procarbazine, and vincristine (R-MPV) chemotherapy regimen (375 mg/m^2^ rituximab, day one; 3.5 g/m^2^ methotrexate, day two; 1.4 mg/m^2^ vincristine, day two; procarbazine 100 mg/m^2^, days two to eight). Rituximab and methotrexate were given every week, while procarbazine and vincristine were given in alternate cycles. Cranial MRI after the induction regimen showed complete resolution of the enhancing parenchymal and optic nerve lesions (Figure 4). Thereafter, the consolidation phase was initiated consisting of two three-week cycles of high-dose cytarabine given at 3 g/m^2^ on day one and day two. Whole brain radiotherapy was not advised due to the patient’s age and risk for neurocognitive decline. After the induction and consolidation therapy, the patient had no recurrence of any visual disturbances and no new neurologic deficits. A follow-up MRI done one month after the consolidation therapy showed no evidence of recurrent intracranial lymphomatous infiltration.

## Discussion

PCNSL can present with various manifestations depending on the location of the tumor. In the majority of cases, the initial symptom manifests as cognitive and behavioral problems, which can lead to a delay in evaluation and diagnosis. It also commonly presents with focal neurologic deficits correlating to the involved brain parenchyma. Visual symptoms rarely present as the initial manifestation of PCNSL as seen in only 4% of cases [4]. Visual problems occur mostly in association with primary intraocular lymphoma (PIOL). PIOL is a specific subset of PCNSL, which mainly affects the retina, vitreous, and optic disc leading to visual disturbances. In some cases, PIOL occurs concurrently with parenchymal lesions of primary CNS lymphoma. Infrequently, the visual disturbance may result from lymphomatous infiltration of the optic nerve.

The optic nerve is rarely involved in both CNS lymphoma and systemic lymphoma. In a study done by Kim et al., optic nerve involvement in lymphoma was classified as (i) primary optic nerve, (ii) optic nerve involvement with CNS disease, (iii) optic nerve involvement with systemic disease, and (iv) optic nerve involvement with PIOL [5]. Although the exact mechanism of lymphoma infiltrating the optic nerve remains uncertain, the authors hypothesized that it may be a consequence of the direct extension of malignant cells, hematogenous spread, dissemination via the CSF, or perineural spread.

It is crucial to differentiate whether the visual symptoms of patients with PCNSL are secondary to an ONI or from an underlying PIOL. The temporal course and severity of the visual deterioration are key factors in differentiating the two. The visual symptoms in PIOL are usually non-disabling with insidious course ranging from one to 48 months. On the other hand, ocular symptoms from ONI occur in a subacute onset with more severe visual impairment [3].

The diagnosis of ONI is mainly established with MRI. Findings such as enlargement of the optic nerve, enhancement of the optic nerve sheath, or presence of tram-track sign are supportive of a lymphomatous infiltration of the optic nerve [5]. However, these findings are non-specific and should be correlated with clinical data. In the presence of a parenchymal CNS lesion, the aforementioned MRI findings strengthen the diagnosis of ONI. In these cases, the onset of subacute severe visual symptoms, with enhancement or enlargement of the optic nerve on MRI, without signs of vitreoretinal lymphoma may be sufficient to establish ONI [3]. In cases when no concomitant parenchymal lesion is seen, CSF cytology may aid in the diagnosis as it can reveal malignant cells. The use of concurrent CSF flow cytometry with CSF cytology has also been proven to increase the detection rate of CNS lymphoma [6].

Management for ONI requires treatment of the underlying PCNSL. Therapeutic options are diverse including surgery, high-dose methotrexate-based chemotherapy regimen, and whole brain radiation therapy [7]. Treatment should employ a multidisciplinary and personalized approach. Visual outcome in patients with ONI depends on the timely initiation of treatment. In a case series done by Ahle et al., which documented seven cases of ONI in PCNSL, all of which had poor baseline visual acuity noted at less than 1/10 (0.1), only two cases had improved visual acuity to 0.3 and 0.6 after chemotherapy [3]. In one of the two improved cases, high-dose corticosteroid therapy was also given before chemotherapy. The improvement was largely attributed to the early initiation of treatment given 10-21 days from symptom onset.

In our case, the patient had a long time from the onset of symptoms to chemotherapy. Steroid therapy, however, was started while awaiting initiation of chemotherapy, which showed subsequent improvement in visual outcome. The time from the onset of symptoms to steroid treatment was 20 days, while the time from the onset of symptoms to chemotherapy was 40 days. Temporization with the use of steroids before chemotherapy helped improve the visual outcome of our patient.

Proper evaluation and treatment of PCNSL are therefore vital in the visual recovery of patients with ONI. Of equal importance is proper monitoring of these cases as it was documented that the prognosis of ONI is poor even with chemotherapy [3]. Due to the small number of cases of ONI published thus far, further studies are needed to better establish the prognosis and outcome of patients with ONI.

## Conclusions

ONI in primary CNS lymphoma is a rare occurrence and presents as a diagnostic challenge. It presents as a subacute onset of severe visual deterioration in the absence of vitreoretinal involvement. Postulated mechanisms of optic nerve involvement include direct tumor cell invasion, hematogenous spread, dissemination through the CSF, and perineural spread. When it is the sole manifestation of the disease, establishing the diagnosis can become difficult as the differential diagnosis is broad including demyelinating disease, infection, and other neoplastic processes. Recognition of clinical symptoms aided by diagnostics for ophthalmologic workup, cranial and orbital MRI, and CSF studies may help establish the diagnosis. Immediate evaluation and treatment with steroid therapy and chemotherapy are crucial for improving the visual outcome of patients with ONI.
